# Evidence for induction of a tumor metastasis-receptive microenvironment for ovarian cancer cells in bone marrow and other organs as an unwanted and underestimated side effect of chemotherapy/radiotherapy

**DOI:** 10.1186/s13048-015-0141-7

**Published:** 2015-03-28

**Authors:** Pranesh M Gunjal, Gabriela Schneider, Ahmed Abdelbaset Ismail, Sham S Kakar, Magda Kucia, Mariusz Z Ratajczak

**Affiliations:** Stem Cell Institute at James Graham Brown Cancer Center, University of Louisville, 500 South Floyd Street, Louisville, KY 40202 USA; Department of Regenerative Medicine, Medical University of Warsaw, Warsaw, Poland

**Keywords:** Prometastatic microenvironment, Metastasis, Inflammation, Side effects of radiochemotherapy, Ovarian cancer

## Abstract

**Background:**

One of side effects of chemotherapy and radiotherapy is the induction of several factors in various tissues and organs that create a pro-metastatic microenvironment for cancer cells that survive initial treatment.

**Methods:**

In the present study, we employed human ovarian cancer cell line A2780 and immunodeficient mice xenogrfat model to test effect of both ibuprofen and dexamethasone to ameliorate the therapy-induced pro-metastatic microenvironment in bone marrow, liver, and lung.

**Results:**

In our studies, we found that total body irradiation or administration of cisplatin increases the metastatic spread of human ovarian cancer cells transplanted into immunodeficient mice compared with animals unexposed to irradiation or cisplatin. Moreover, conditioned media harvested from irradiated murine bone marrow, lung, and liver chemoattracted human ovarian cancer cells, and this chemotactic activity was inactivated by heat, suggesting a major involvement of peptide or peptide-bound chemoattractants. We also observed that human ovarian cancer cells proliferate better if exposed to cell debris harvested from irradiated murine bone marrow. Finally, the pro-metastatic microenvironment in mice induced by radio- or chemotherapy was significantly ameliorated if animals were treated at the time of radiotherapy administration with non-steroid (ibuprofen) or steroid (prednisone) anti-inflammatory drugs.

**Conclusions:**

In summary, we propose that a radiochemotherapy-induced, pro-metastatic microenvironment plays an important role in the metastasis of cancer cells that are resistant to treatment. Such cells have characteristics of cancer stem cells and are highly migratory, and simple, intensive, anti-inflammatory treatment by non-steroid agents to suppress induction of pro-metastatic factors after radiochemotherapy would be an interesting anti-metastatic treatment alternative.

**Electronic supplementary material:**

The online version of this article (doi:10.1186/s13048-015-0141-7) contains supplementary material, which is available to authorized users.

## Introduction

After surgical removal of tumor tissue, chemotherapy and radiotherapy are the most efficient treatment modalities employed in clinical oncology. Unfortunately, anti-cancer treatment is usually associated with toxicity to non-tumor cells, resulting in different degrees of damage to tissues and organs [1,2]. There are well-known side effects of chemotherapy and radiotherapy that are mainly related to the toxicity to, and impaired function of, vital organs; however, the induction by these therapies of the expression of several factors in various organs that create a pro-metastatic microenvironment, is, surprisingly, not widely acknowledged.

In support of the reality of this effect, it is very well known that normal hematopoietic stem cells, after infusion into a host following myeloablative radiochemotherapy, home efficiently to bone marrow (BM) in response to several chemotactic factors upregulated in the BM microenvironment [3-5]. To explain this phenomenon, we hypothesized that toxic damage to BM and other organs as a result of radio and/or chemotherapy treatment of malignancies leads to upregulation of factors that attract normal circulating stem cells for regeneration but, unfortunately, also provide chemotactic signals to cancer cells that survive the initial treatment [1,6,7]. These cells may then establish tumor metastases.

In support of toxic damage as a driver of metastasis, we as well as others have demonstrated that exposure of mice to irradiation [8,4], cyclophosphamide [8,9], or vincristine [6,7] upregulates the levels of several chemokines and growth factors, such as stromal-derived factor 1 (SDF-1), hepatocyte growth factor/scatter factor (HGF/SF), vascular endothelial growth factor (VEGF), and monocyte chemotactic protein 1 (MCP-1), in the BM microenvironment [8-11]. We predicted that a similar response would accompany the toxic effects of chemotherapy or radiotherapy in other sensitive organs, including liver and lung [1,6,7].

In the present study, we report that conditioned medium (CM) from irradiated BM, lung, and liver chemoattracts human ovarian cancer cells in vitro. Next, by employing a short in vivo tumor cell tissue-seeding assay, we observed that increased numbers of human cancer cells seed (metastasize) to the BM and other organs in SCID-Beige immunodeficient mice after irradiation or exposure to chemotherapy. In addition, we observed that human cancer cells expanded more strongly in culture in the presence of a feeder layer of cell debris from damaged or dying human BM cells. Finally, we were able to (1) ameliorate the in vitro migration of cancer cells to CM harvested from irradiated tissues and (2) decrease seeding of in vivo-injected human cells into various organs in irradiated or chemotherapy-exposed mice by reducing the pro-inflammatory response induced by radiochemotherapy, due to administering either of the non-steroid anti-inflammatory drugs ibuprofen or dexamethasone.

We propose that a radiochemotherapy-induced pro-metastatic microenvironment, which plays an important but underappreciated role in the metastasis of cancer cells to bone and other organs (lungs or liver), can be ameliorated by a simple, intensive treatment by non-steroid and steroid anti-inflammatory agents to suppress induction of pro-metastatic factors by radiochemotherapy.

## Material and methods

### Cell lines

The A2780 ovarian epithelial cancer cell line was maintained in Roswell Park Memorial Institute (RPMI) medium 1640, containing 10% FBS, 100 U/ml penicillin, and 10 μg/ml streptomycin. Cells were cultured in a humidified atmosphere of 5% CO_2_ at 37°C, and the medium was changed every 48 hours.

### Peritoneal tumor formation in mice

All procedures involving animals and their care were approved by the Institutional Animal Care and Use Committee, according to the guidelines of the association for Assessment and Accreditation of Laboratory Animal Care of the University of Louisville (Louisville, KY, USA).

Human ovarian cancer cells (2.5 × 10^6^) were inoculated into the peritoneal cavity of SCID-Beige inbred mice, and seven mice were used for each group. The mice were monitored for peritoneal swelling and any adverse effects and euthanized after 3–5 weeks of cell inoculation. Visible tumor nodules were excised and collected and were measured for size.

**Figure 1:**
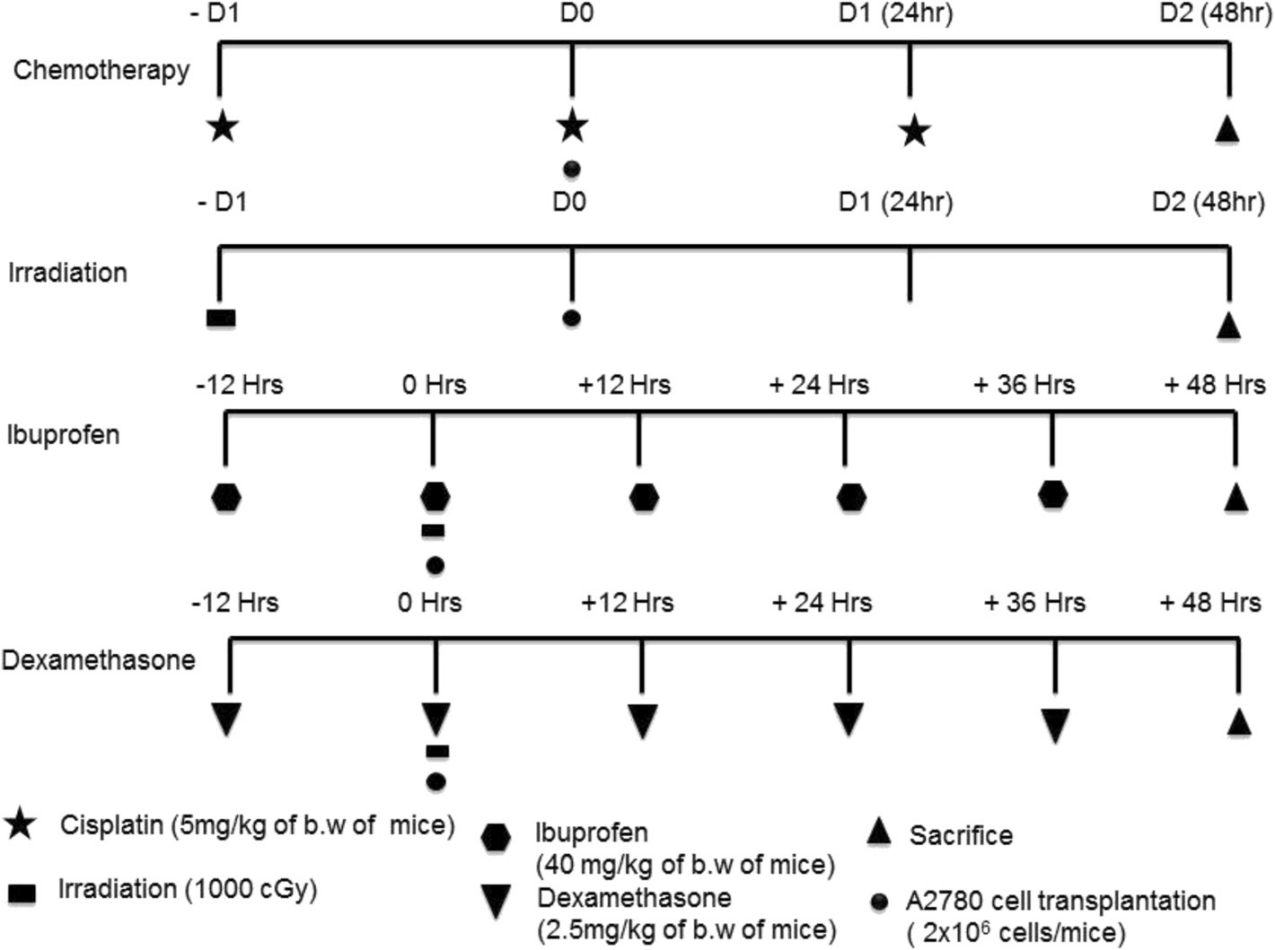
**Radiochemotherapy and anti-inflammatory drug injection strategy.** SCID-Beige inbred mice received a chemodrug (cisplatin) for three consecutive days, −D1, D0, and D1, and A2780 cells were transplanted 24 hrs after the first cisplatin injection. Similarly, SCID-Beige inbred mice underwent irradiation at 1000 cGy on –D1, prior to A2780 cell injection. SCID-Beige inbred mice also received anti-inflamatory drugs (ibuprofen or dexamethasone) for five consecutive injections (−12 hrs, 0 hrs, +12 hrs, +24 hrs, +36 hrs) and underwent irradiation at 1000 cGy at 0 hrs, followed immediately by A2780 celltransplantation. All drugs were injected at doses explained in the figure. All mice were sacrificed 48 hours after the injection of A2780 cells, and bone marrows, spleens, livers, and lungs were collected. The presence of ovarian cells (i.e., murine–human chimeras) was evaluated by the level of human α-satellite DNA expression.

### Injection of A2780 cells into immunodeficient mice

To evaluate the in vivo metastatic behavior of A2780 cells, cells were injected intravenously (i.v.; 2 × 10^6^ per mouse) into severe combined immunodeficient (SCID)-Beige inbred mice and divided into four groups (five mice per group). Group i) control (non-irradiated mice), ii) irradiated with 1000 cGy 24 hours prior to injection of A2780 cells, iii) control (untreated and non-irradiated), and iv) injected with cisplatin (5 mg/kg of body weight of mice) anticancer drug.

In a second experiment, mice were injected with an anti-inflammatory drug prior to irradiation (five mice per group). Mice were grouped as i) control (untreated and non-irradiated), ii) irradiated with 1000 cGy 24 h prior to injection of A2780 cells, iii) injected with ibuprofen (40 mg/kg of body weight of mice) only, iv) injected with ibuprofen (40 mg/kg of body weight of mice) and irradiated with 1000 cGy, v) injected with dexamethasone (2.5 mg/kg body weight of mice) only, and vi) injected with dexamethasone (2.5 mg/kg body weight of mice) and irradiated with 1000 cGy. In both experiments, all control animals were injected with A2780 cells only. Experimental strategies are shown in (Figure 1).

Bone marrows, spleens, livers, and lungs were removed 48 hours after injection of these cells, and the presence of ovarian cells (i.e., murine–human chimeras) was evaluated by the level of human α-satellite DNA expression. DNA was amplified in the extracts isolated from BM-, spleen-, liver-, and lung-derived cells using real-time PCR. Briefly, DNA was isolated using the QIAamp DNA Mini kit (Qiagen), and human satellite and murine β-actin DNA levels was detected by means of real-time PCR using an ABI Prism 7500 Sequence Detection System. A 25-μl reaction mixture containing 12.5 μl SYBR Green PCR Master Mix, 300 ng DNA template, forward (5′-ACC ACT CTG TGT CCT TCG TTC G-3′) and reverse (5′-ACT GCG CTC TCA AAA GGA GTG T-3′) primers for the α-satellite, and forward (5′-TTC AAT TCC AAC ACT GTC CTG TCT -3′) and reverse (5′- CTG TGG AGT GAC TAA ATG GAA ACC -3′) primers for the β-actin were used. The C_t_ value was determined as described previously [7], and the number of human cells present in the murine organs (the degree of chimerism) was calculated from the standard curve obtained by mixing different numbers of human cells with a constant number of murine cells. In long-term experiments, cells (2.5 × 10^6^ per mouse) were inoculated intraperitoneally into SCID-Beige inbred mice (seven mice per group). Four weeks later, the mice were sacrificed for evaluation of the A2780 cells present in BM, spleen, liver, and lung. Detection of human cells was performed as described below (Additional file 1: Figure S1, panel A).

### Flow cytometric analysis of HLA expression

BM cells were harvested from mice at day 30 following transplantation of A2780 cells. Mice were grouped (four mice per group) according to treatment: i) no irradiation or ii) irradiation with 1000 cGy 24 hours prior to A2780 cell transplantation. BM cells were then washed twice with sterile PBS and incubated with 1X RBC lysis solution, and the intact cells were stained for human leukocyte antigen (HLA) Additional file 1: Figure S1, panel B. Staining was performed in PBS with 2% fetal bovine serum (FBS, Invitrogen, Carlsbad, CA), at 4°C for 30 min. Cells were subsequently washed, resuspended, and analyzed using an LSR II flow cytometer (BD Biosciences). At least 1.5 × 10^6^ events were acquired from each sample, and FlowJo software was used for analysis (Tree Star, Ashland, OR).

### Preparation of conditioned media

Pathogen-free C57BL/6 mice (7–8 weeks old) were used for these experiments. Mice (four per group) were irradiated with 1000 cGy, and 24 hours later, bone marrow, liver, and lung were isolated. Conditioned medium (CM) was obtained after 3 hours of incubation of BM, liver, or lung cells (mechanically homogenized 30x using a syringe) in RPMI at 37°C. After centrifugation, the supernatant was used for further experiments. In studies with anti-inflammatory drugs, mice were injected intraperitoneally with ibuprofen (40 mg/kg) or dexamethasone (2.5 mg/kg) prior to irradiation with 1000 cGy. Twenty-four hours later, organs were isolated, and CM from various organs was prepared as described above.

### Chemotaxis assay

Chemotaxis assays were performed in a modified Boyden’s chamber with 8-μm pore polycarbonate membrane inserts (Costar Transwell; Corning Costar, Lowell, MA, USA) as described previously [7]. In brief, cells were detached with 0.05% trypsin and seeded into the upper chamber of an insert at a density of 5 × 10^4^ in 110 μl. The lower chamber was filled with pre-warmed CM harvested from the different organs from irradiated SCID-Beige mice at 1000 cGy. Medium supplemented with 0.5% BSA was used as a negative control, and medium supplemented with 10% FBS was used as a positive control. After 48 hours, the inserts were removed from the Transwell supports. The cells that had not migrated were scraped from the upper membrane with a cotton swab, and the cells that had transmigrated to the lower side of the membrane were fixed and stained with HEMA 3 (Protocol, Fisher Scientific, Pittsburgh, PA) and counted on the lower side of the membrane using an inverted microscope.

### Temperature effect on BM CM

CM samples from irradiated mouse BM were subjected to a range of temperatures. In particular, they were: i) incubated for 30 min at 37°C, 42°C, 56°C, or 90°C and ii) subjected to three frost–defrost cycles, which were carried out by incubating these CM samples at −80°C and subsequently in a water bath at 37°C. The samples were then directly used for chemotaxis experiments, and any residual samples were stored at −80°C for further use.

### Molecular centrifugation of BM CM

CM samples from irradiated mouse BM were centrifuged at 3000 × g for 30 min in Centricon centrifugal filter devices (Millipore) until the entire sample had passed through the column. These devices were used for the separation of proteins and peptides, covering a range of molecular-weight cutoffs (10, 30, 50, and 100 kDa). CM samples filtered and concentrated using different molecular-weight cutoffs were then used for chemotaxis assays with A2780 cells.

### Invasive potential of ovarian cancer cells grown on dying or damaged bone marrow cells

C57BL/6 GFP mice were grouped (three per group) as non-irradiated or irradiated (with 1000 cGy). Twenty-four hours later, bone marrow, liver, and lung were isolated, and CM samples were obtained from these organs, as described above. After 3 hours, cells flushed from bone marrow were then co-cultured with A2780 cells (1000 cells/well in 24-well plates). Both BM cells and CM samples from other organs were analyzed as a growth-supporting medium for A2780 cells. Forty eight hours later, A2780 cell clusters were counted and photographed under a fluorescence microscope, and GFP-expressing BM cells were also counted by FACS.

### Statistical analysis

All results were presented as mean ± SD. Statistical analysis of the data was performed using the nonparametric Mann–Whitney test (animal studies) and Student’s t test for unpaired samples by using Graph Pad Prism software. P < 0.05, P < 0.005 and P < 0.0005 were considered significant.

## Results

### Increased seeding efficiency of human ovarian cancer cells in a mouse xenograft model after chemotherapy and irradiation

**Figure 2:**
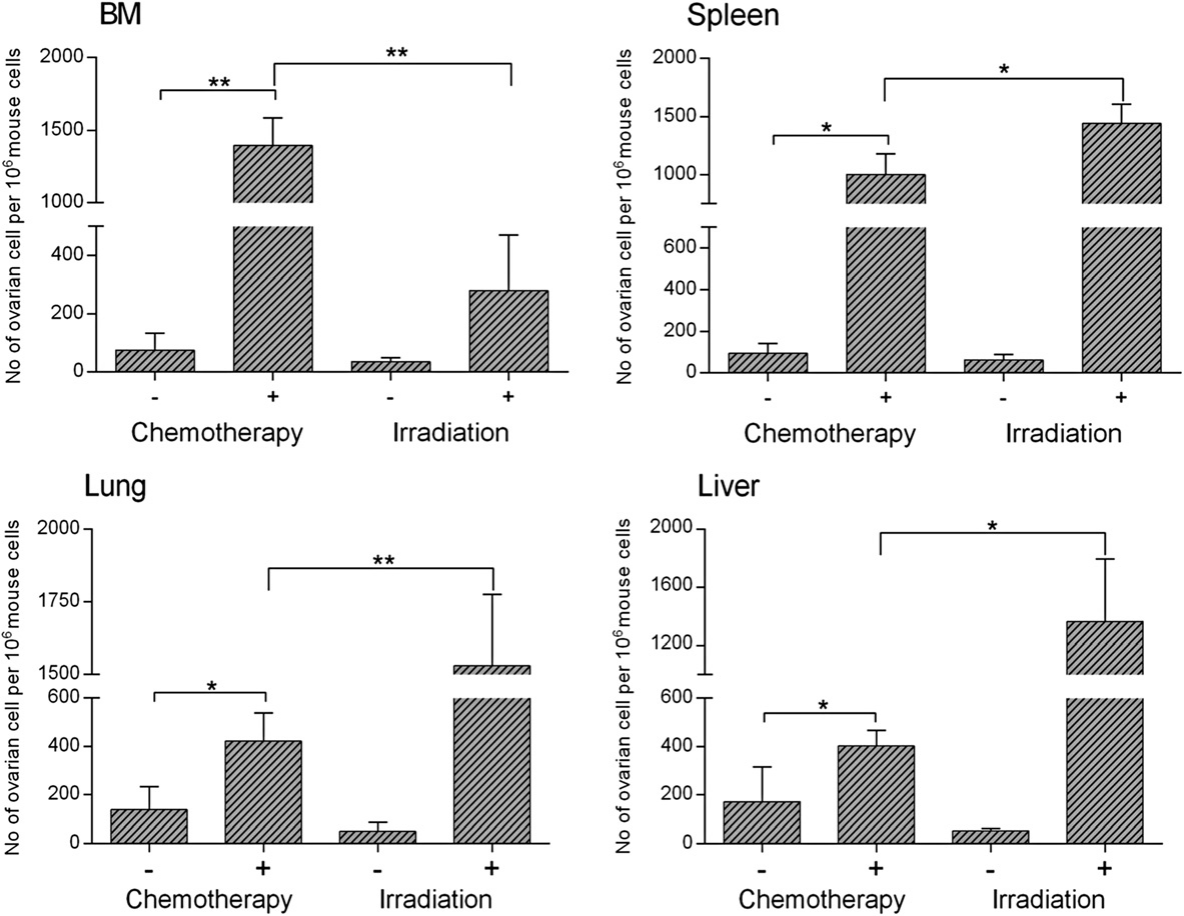
**Chemotherapy and irradiation create a pro-metastatic microenvironment in various murine organs that chemoattract A2780 cells.** qRT-PCR detection of A2780 human ovarian cancer cells in bone marrow (BM), liver, lung, and spleen after irradiation (1000 cGy) and chemotherapy (cisplatin). Five mice were employed per group. The results from two independent experiments are combined and shown as mean ± SD. *p < 0.05 or **p < 0.005 compared with control (organs neither irradiated nor received cisplatin).

To test the hypothesis that chemotherapy and irradiation induce a pro-metastatic microenvironment in various organs, immunodeficient SCID-Beige mice were exposed to three doses of cisplatin (5 mg/kg bw) administered i.p. on days −1, 0, and +1 after i.v. injection of A2780 human ovarian cancer cells (2 × 10^6^ cells/mouse). In parallel, experimental mice were irradiated with 1000 cGy on day −1 before i.v. injection of ovarian cancer cells at the dose described above. Control mice were neither exposed to cisplatin nor irradiated. Mice were sacrificed 48 hours after cell injection, and cell suspensions from bone marrow, spleen, lung, and liver were prepared and DNA extracted for real-time PCR analysis of human Alu sequences. Based on standard curves, different levels of Alu sequence expression were converted to the number of human cells present in murine tissues. Figure 2 shows that we were able to detect a significant increase in human ovarian cancer cells present in BM, liver, spleen, and lung in mice that were exposed to cisplatin or were irradiated.

### Anti-inflammatory treatment at the time of irradiation reduces the metastatic spread of human ovarian cancer cells in immunodeficient mice

**Figure 3:**
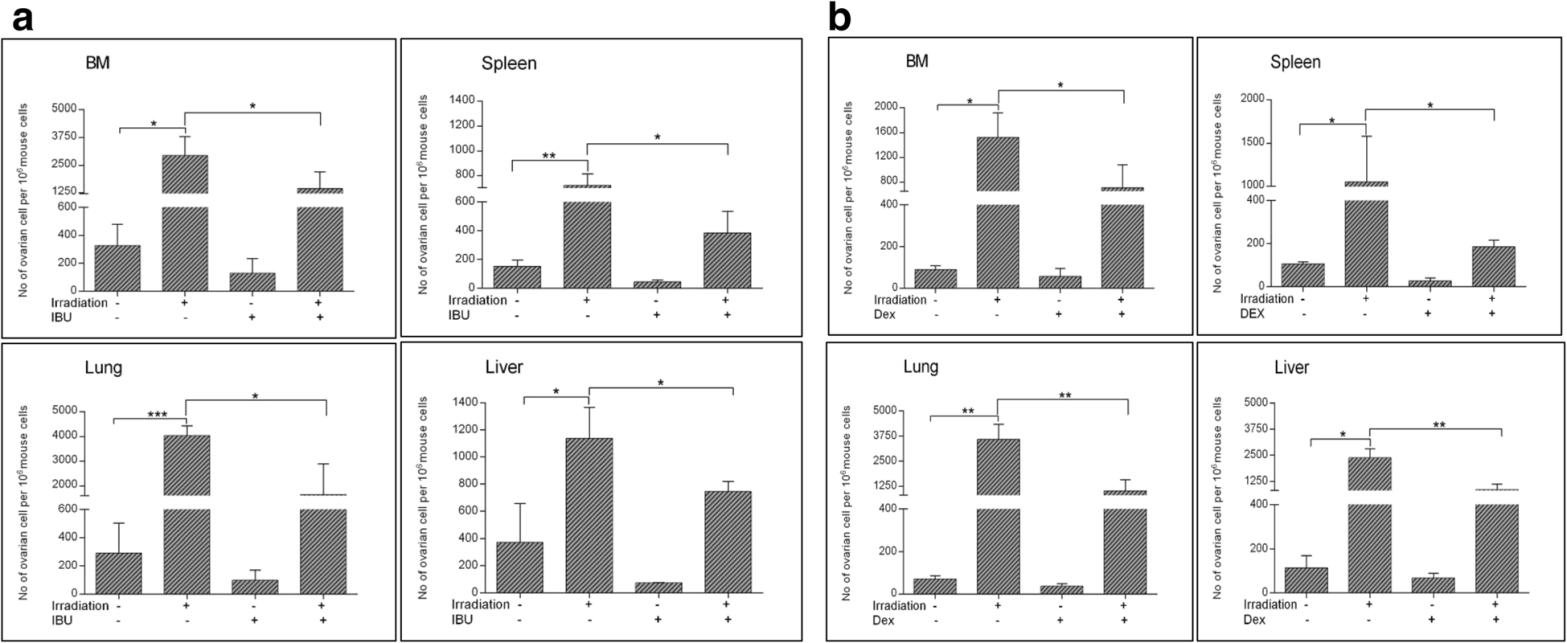
**Anti-inflammatory treatments inhibit the metastatic spread of A2780 cells.** Ibuprofen **(Panel a)** and dexamethasone **(Panel b)** significantly decrease the metastatic spread of A2780 human cancer cells into bone marrow (BM), liver, lung, and spleen of SCID-Beige inbred mice. In these experiments, five mice were used per group, and the results from three independent experiments for each anti-inflammatory drug are shown as mean ± SD. *p < 0.05, **p < 0.005, or ***p < 0.0005 for the group receiving irradiation (1000 cGy) compared with the control group (no irradiation) and for the groups receiving irradiation and an anti-inflammatory drug compared with the group receiving irradiation alone.

Based on the observation that irradiation results in damage to several organs and induces a pro-metastatic microenvironment that may promote the spread of cancer cells, we tested whether administration of anti-inflammatory drugs at the time of irradiation would inhibit the spread of ovarian cancer cells to different organs. In this experiment we administered ibuprofen (40 mg/kg bw) or dexamethasone (2.5 mg/kg) to mice at −12 hrs and subsequently at 0, +12, +24 and +36 hrs after irradiation. Employing ibuprofen or dexamethasone as an anti-inflammatory treatment showed a significant effect in ameliorating metastatic spread of ovarian cancer cells that were injected i.v. with A2780 cells 3 hours after irradiation (Figure 3 panels A and B).

### Human ovarian cancer cells respond to a chemotactic gradient of factors released from irradiated organs

**Figure 4:**
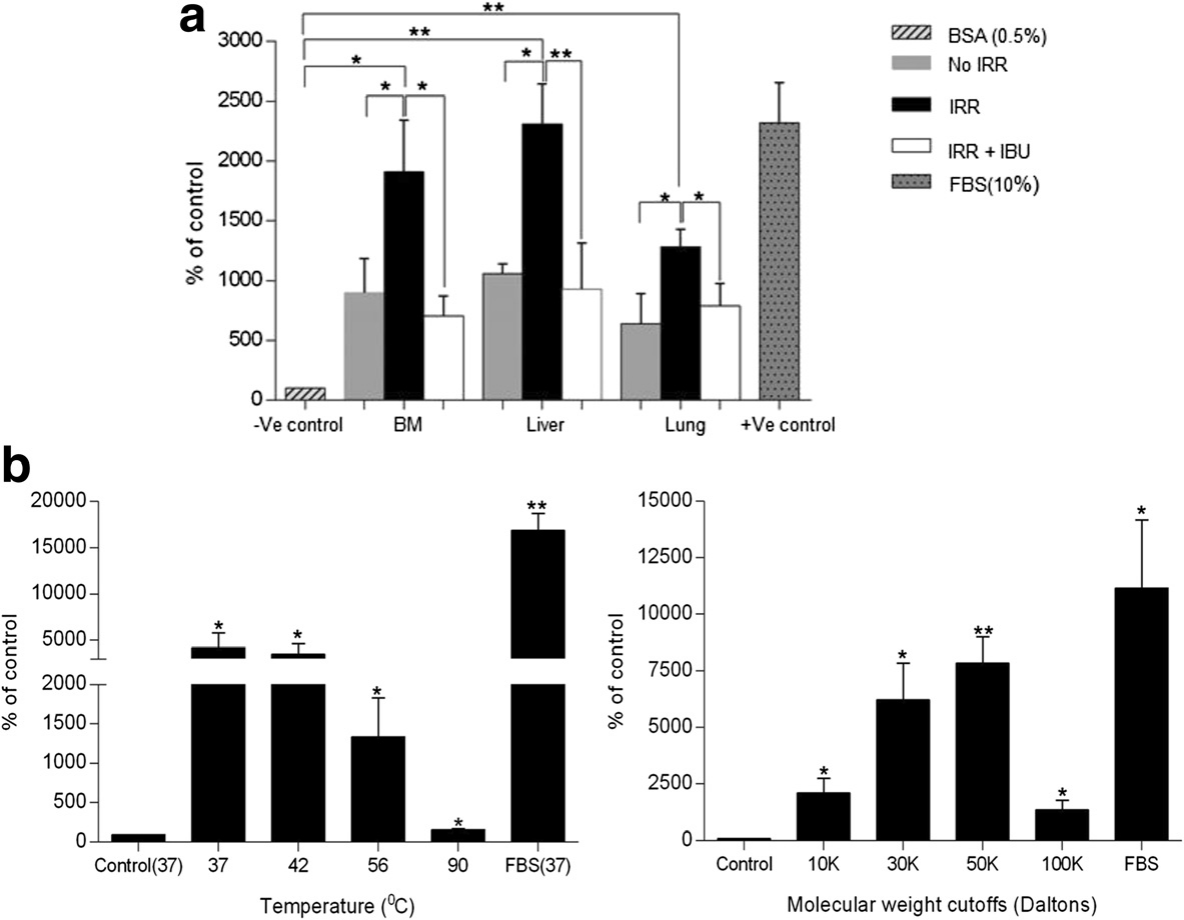
**Conditioned media (CM) derived from irradiated organs are potent chemoattractants for A2780 cells. a**. Chemotaxis of A2780 cells across Transwell membranes in response to CM harvested from BM, liver, and lung of irradiated and ibuprofen-treated SCID-beige inbred mice. **b.** Chemotaxis of A2780 cells across Transwell membranes in response to irradiated BM CM samples subjected to heat inactivation at different temperatures (left panel) and derived from molecular filtration by using Centricon centrifugal filtration devices (right panel). The chemotaxis assay was performed at least three times in duplicate, with similar results. Results are presented as mean ± SD, with a statistical significance *p < 0.05 or **p < 0.005 relative to the control (cells stimulated with RPMI 0.5% BSA medium).

Next, we collected conditioned media (CM) from organs harvested from irradiated SCID-Beige mice. Animals were irradiated with 1000 cGy, and 24 hours later, organs were removed and cell suspensions were incubated for 3 hours. Finally, CM samples were prepared by removing cells and cell debris by high-speed centrifugation and employed as a source of chemoattractants that were placed in the lower chambers in Transwell migration assays. In parallel, we prepared CM samples from irradiated mice treated with ibuprofen. We observed a significant chemotactic effect of CM harvested from irradiated BM liver, and lung and a significant decrease in chemotactic activity in CM prepared from organs isolated from ibuprofen-treated irradiated animals (Figure 4A).

Based on these results, we became interested in pinpointing the molecular characteristics of the chemotactic factors present in CM harvested from BM. Figure 4B shows an initial analysis of these CM sample by employing heat inactivation (left panel) and molecular centrifugation (right panel). The results indicate that chemotactic factors are heat sensitive, which suggests a peptide- or protein-associated structure, and are in the range of 30–50 kDa in size. We are currently trying to identify these factors by employing mass spectrophotometry analysis.

### Lethally irradiated BM tissue supports growth of human cancer cells

**Figure 5:**
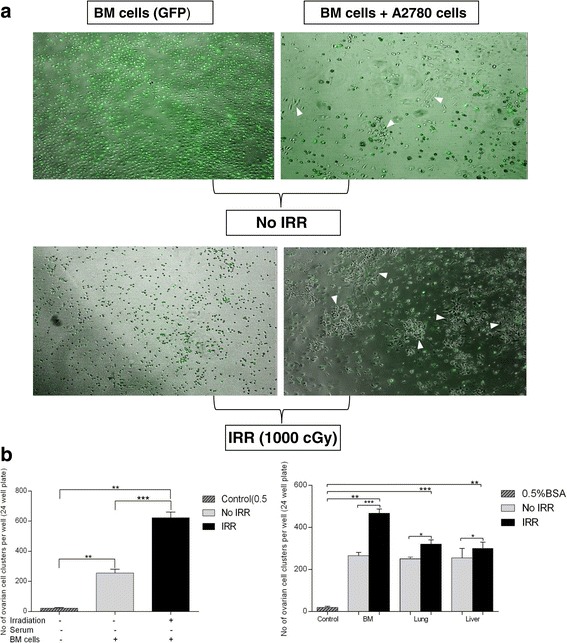
**Damaged bone marrow cells and CM harvested from organs from irradiated mice support A2780 cell growth in vitro. a**. Proliferation and expansion of A2780 cells co-cultured with irradiated BM cells from C57BL/6 GFP mice for 48 hours, showing that irradiation causes lethally damaging effects to BM cells that result in the release of growth- and metastasis-promoting factors in vitro. Non-irradiated BM cells showed little or no effect on the growth of A2780 cells (upper panel). On the other hand, irradiated BM cells support the rapid and spontaneous growth of A2780 cell clusters (lower panel). **b**. Microscopic count of A2780 cell clusters found per well (24-well culture plate) after 48 hours of co-culture with irradiated and non-irradiated BM cells (left panel). Microscopic count of A2780 cell clusters after 48 hours of co-culture with CM samples harvested from BM, lung, and liver from irradiated and non-irradiated mice. (right panel). This co-culture experiment was repeated twice. In each experiment, six mice were employed per group (n = 3, no-irradiation control; n = 3, irradiation at 1000 cGy). Microscopic counts were made in triplicate from the wells, with significant results. Results are presented as means ± SD, with a statistical significance *p < 0.05, **p < 0.005, or ***p < 0.0005 relative to the control (A2780 cells cultured in RPMI 0.5% BSA medium).

Based on the findings that irradiated BM chemoattracts human ovarian cancer cells, we tested another hypothesis: that damaged BM tissue supports expansion of metastasizing cancer cells. To address this issue, transgenic green immunofluorescence (GFP^+^) mice were lethally irradiated with 1000 cGy. BM cells were recovered 24 hrs later from bone and plated without washing into 24-well culture plates. After 3 hours, A2780 cells were added at 10^3^/well. Established in this way, co-cultures of irradiated murine BM cells and human A2780 cancer cells were evaluated after 48 hours. Figure 5A shows representative images of the effect of non-irradiated (upper panel) and irradiated (lower panel) BM cells on the clonal expansion of human ovarian cancer cells. One can see that debris from irradiated BM cells increases proliferation of A2780 cells (right panel). To quantify this data, the number of A2780 cell clusters was counted in wells containing non-irradiated (control) and irradiated BM cells (Figure 5B, left panel). In parallel experiments (Figure 5B, right panel), we also evaluated the effect of CM harvested from non-irradiated and irradiated BM, lung, and liver on proliferation of A2780 cells and observed a similar positive effect on human cancer cell growth.

## Discussion

The coincidence between inflammation and tumorogenesis is well established as well as the fact that cancer frequently develops in tissues exposed to chronic inflammation [12,13], which orchestrates the microenvironment around growing tumors, contributing to proliferation, survival, and migration of malignant cells [12-14]. Therefore, it is not surprising that both non-steroid and steroid anti-inflammatory drugs are employed in cancer patients in some protocols [15,16].

Specifically, non-steroid, anti-inflammatory drugs such as the cyclooxygenase-1 and −2 inhibitor ibuprofen or the more potent and selective COX-2 inhibitor celecoxib appear to reduce the risk of developing cancer and may inhibit prostaglandin- and thromboxane-dependent cell division, metastasis, and angiogenesis and promote the apoptosis of malignant cells [17,18]. On the other hand, synthetic corticosteroids, such as dexamethasone, are also used in oncology in patients undergoing chemotherapy to counteract certain side effects, such as emesis, or to counteract edema in brain tumors [19,20]. Dexamethasone is also used as a direct chemotherapeutic agent in certain hematological malignancies [21,22].

In the present study, we employed both ibuprofen and dexamethasone to ameliorate the therapy-induced pro-metastatic microenvironment in bone marrow, liver, and lung. Based on our experimental models and results, we propose a potential application of anti-inflammatory treatment for cancer patients by administration of a COX-2 inhibitor or dexamethasone at the time of radiochemotherapy to reduce the metastatic spread of cancer cells to different organs. This strategy is based on the finding that exposure of mice to γ-irradiation or systemic administration of cisplatin induces a pro-metastatic microenvironment in BM, liver, spleen and lung (Figures 2 and 3). The salient observation of our work is that administration of ibuprofen or dexamethasone at the time of radiochemotherapy inhibits the spread of human ovarian cancer cells to several organs of immunodeficient mice. This is relatively a simple approach that is effective at significantly decreasing the seeding efficiency of human malignant cells to several organs after they are injected intravenously. This essay has been developed by our group to study early metastatic spread of tumor cells [6,7]. However, we are aware that more experiments are needed to evaluate metastatic potential of cancer cells over time. We also show that ovarian cancer cells grow and expand much better when exposed to debris from BM-irradiated cells. Again, these studies performed on established tumor cell line need further clarification in real clinical settings employing patient cancer cells that survived radio-chemotherapy treatment.

It is well known that metastasis is responsible for more than 90% of cancer-associated mortality [23,24] and is a multistep process in which induction of a pro-metastatic microenvironment within tumors and at distant locations plays a crucial role. Chemotactic factors emerging in damaged tissues may induce an undirected motility of cancer cells, described as chemokinesis [25], or a directed migration, known as chemotaxis [26]. In response to chemokinesis and/or chemotaxis, cancer cells leave a primary tumor, infiltrate surrounding tissues, and may enter the lymphatics or peripheral blood to metastasize to remote organs [1,6-8]. Several peptide-based and non-peptide-based factors, such as bioactive lipids or extracellular nucleotides, have been described as inducing the motility of cancer cells [4,6,7,27]. On the other hand, enhanced migratory properties are one of the key features of stem cells [28]. The comparatively strong resistance of cancer therapy supports the concept of presence of cancer stem cells [29-32]. These cells would survive treatment and are most likely responsible for malignant spread after the initial reduction of the primary tumor. Since metastasis and tumor spread is a major factor limiting survival of cancer patients, the clinical need to prevent or target metastasis is a therapeutic priority in clinical oncology.

It is well known that tumors respond to several pro-metastatic chemotactic factors, and therefore it would be difficult to inhibit their metastatic potential by inhibiting just one or even a few receptor axes [4,6,7,33]. For example, in a model of metastatic rhabdomyosarcoma, we previously demonstrated that exposure to irradiation [4,6,7] or chemotherapy [6-8] of several organs upregulates the levels of chemokines and growth factors, such as SDF-1, HGF/SF, VEGF, and MCP-1, in addition to bioactive lipids such as S1P, C1P, LPA, and LPC, which are endowed with strong chemotactic activities with respect to normal as well as malignant cells [3,6,7]. As in chemotherapeutics, exposure to irradiation leads also to the release of several alarmines (e.g., ATP and UTP) from the damaged cells (manuscript in preparation). Moreover, in several well-controlled animal experimental models, it has been demonstrated that blockade of CXCR4 [4,33,34], CXCR7 [35], or c-Met [4,36] receptors by employing small-molecule inhibitors; downregulation of these receptors by shRNA strategies [27,37]; *in vivo* administration of blocking antibodies against SDF-1 [38] or MCP-1 [39,40]; or application of S1P-binding aptamers [6] significantly diminishes chemotherapy- or radiotherapy-related dissemination of tumor cells to various organs. Since it is impossible to target all these pro-metastatic factors at the same time, it is obvious that future anti-metastatic drugs must depend on potent molecules that interfere with migration and adhesion processes of cancer cells downstream of the surface receptors for these pro-metastatic factors.

However, the aim of our current work was not to identify particular factors involved in radiochemotherapy-induced metastatic spread of cancer cells. but rather to broadly characterize their molecular properties. Our preliminary experiments, performed in a model of human ovarian cancer, indicate the involvement of temperature-sensitive factors that are present in the 30–50-kDa fraction of normal serum. While this fraction is most likely to contain a peptide-based chemoattractant (s), we cannot exclude the possibility that it may contain certain bioactive lipids that are associated with proteins. Further studies will address this issue. We are also aware that the metastatic spread of cancer cells after radiochemotherapy could also be promoted by other mechanisms. One of these mechanisms could be direct toxicity to the endothelial wall, which affects the integrity of the endothelial barrier, and may facilitate seeding of cancer cells into damaged organs through the disrupted endothelium [9]. Another possibility is that membrane fragments (e.g., exosomes or microvesicles) have been shown in several animal models to be endowed with chemotactic properties [41,42]. Furthermore, we must remember that our results were obtained with a human ovarian cancer cell line, and cells from other tumors may respond differently to a panel of chamoattractants.

In conclusion, we propose that a radiochemotherapy-induced pro-metastatic microenvironment plays an important role in the metastasis of cancer cells that are resistant to treatment. Such cells possess characteristics of cancer stem cells and are highly migratory, and a simple, intensive treatment with anti-inflammatory agents to suppress induction of pro-metastatic factors after radiochemotherapy is an interesting treatment alternative. However, this hypothesis requires further dose-optimization studies and validation in appropriate clinical trials. Finally, as we have also demonstrated in a model of irradiated BM, cell debris from organs damaged by radiochemotherapy may support expansion of cancer cells and could provide an underappreciated “fertile soil” for metastasizing cancer cells, as suggested in the well-known seed and soil hypothesis of cancer metastasis [43].

## Additional file

Additional file 1: Figure S1. Intraperitoneal murine model of A2780 cell metastasis. A. Metastatic behavior measured by qRT-PCR detection of human ovarian cancer cells (A2780) in various organs on day 30 after intraperitoneal injection into SCID-beige inbred mice. Bilateral ovarian tumors found in mice transplanted with A2780 cells (right box) compared with control mice (left box). In this experiment, seven mice were employed per group, and results are presented as mean ± SD, with a statistical significance *p < 0.05 or **p < 0.005 relative to the control mice (untreated with A2780 cells). B. FACS detection of human leukocyte antigen-positive (HLA^+^) cells in bone marrow (BM) harvested from intraperitoneally injected and irradiated (1000 cGy) SCID-Beige inbred mice on day 30. In this experiment, four mice were employed per group, and results are presented as means ± SD, with a statistical significance *p < 0.05 or **p < 0.005 relative to the control mice (no irradiation).
