# Supplementary figures and images for: Evidence for induction of a tumor metastasis-receptive microenvironment for ovarian cancer cells in bone marrow and other organs as an unwanted and underestimated side effect of chemotherapy/radiotherapy
Source: J Ovarian Res. 2015 Mar 28;8:20. doi: 10.1186/s13048-015-0141-7 (PMC4425926; doi:10.1186/s13048-015-0141-7)

## Slide 1
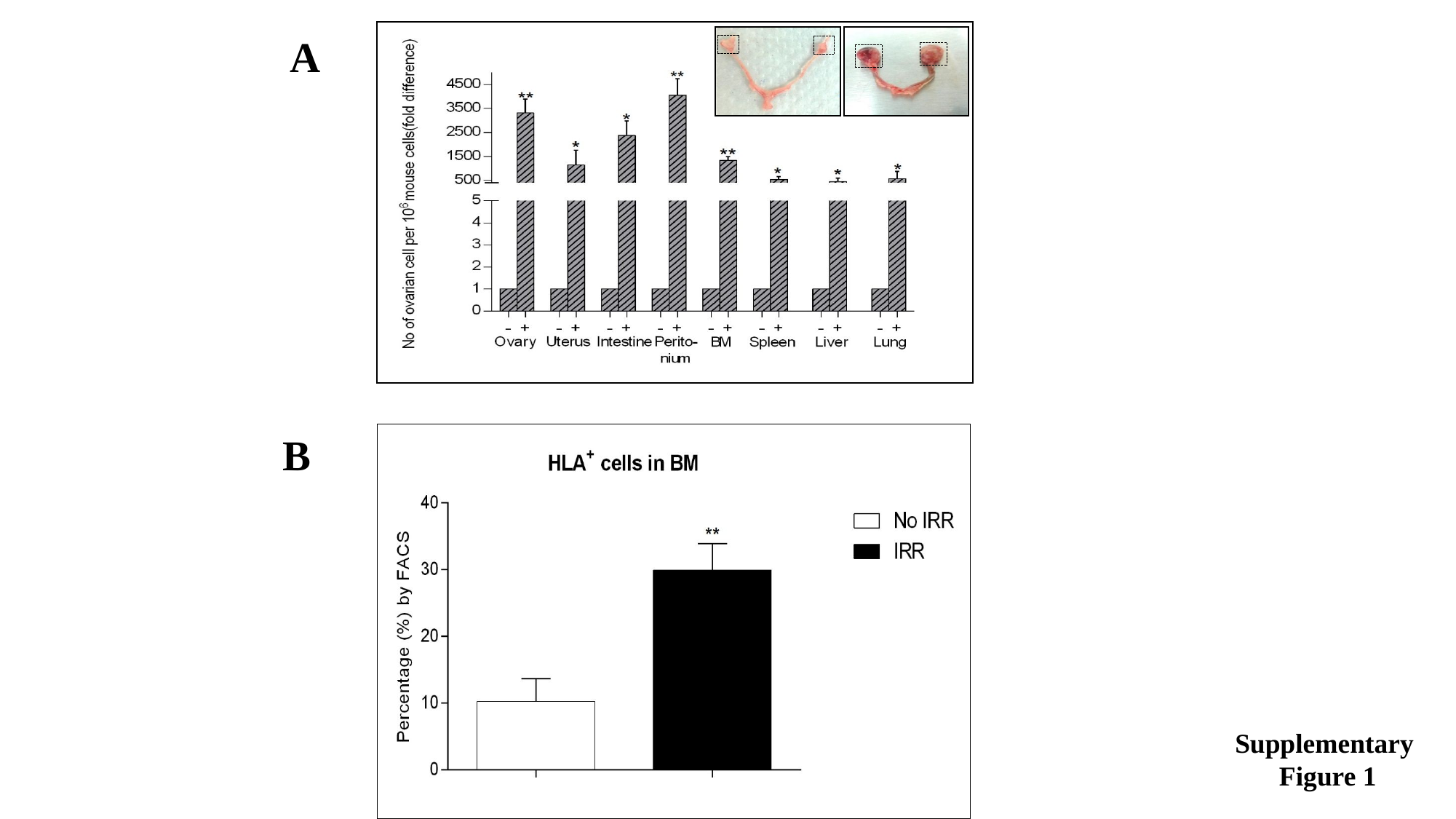

A
B
Supplementary
Figure 1

Supplement: Additional file 1: Figure S1. — Intraperitoneal murine model of A2780 cell metastasis. A. Metastatic behavior measured by qRT-PCR detection of human ovarian cancer cells (A2780) in various organs on day 30 after intraperitoneal injection into SCID-beige inbred mice. Bilateral ovarian tumors found in mice transplanted with A2780 cells (right box) compared with control mice (left box). In this experiment, seven mice were employed per group, and results are presented as mean ± SD, with a statistical significance *p < 0.05 or **p < 0.005 relative to the control mice (untreated with A2780 cells). B. FACS detection of human leukocyte antigen-positive (HLA+) cells in bone marrow (BM) harvested from intraperitoneally injected and irradiated (1000 cGy) SCID-Beige inbred mice on day 30. In this experiment, four mice were employed per group, and results are presented as means ± SD, with a statistical significance *p < 0.05 or **p < 0.005 relative to the control mice (no irradiation). [file 13048_2015_141_MOESM1_ESM.pptx]
